# Carbohydrate Mouth Rinse Increases High but Not Low Intensity Repetitions to Failure in Resistance-Trained Males

**DOI:** 10.3390/nu14040875

**Published:** 2022-02-19

**Authors:** Raci Karayigit, Mustafa Can Eser, Fatih Gur, Cengizhan Sari, Ladislav Cepicka, Tomasz Gabrys

**Affiliations:** 1 Faculty of Sport Sciences, Ankara University, Gölbaşı, Ankara 06830, Turkey; mceser@ankara.edu.tr; 2 Faculty of Sport Sciences, Pamukkale University, Pamukkale, Denizli 20000, Turkey; fatihgur@pau.edu.tr; 3 Faculty of Sport Sciences, Muş Alparslan University, Muş 49250, Turkey; cengizhansarii7@gmail.com; 4 Sport Centrum Faculty of Pedagogy, University of West Bohemia, 301 00 Pilsen, Czech Republic; lcepicka@ktv.zcu.cz (L.C.); tomaszek1960@o2.pl (T.G.)

**Keywords:** muscular endurance, ergogenic aids, mouthwash, supplement, high intensity, rinsing

## Abstract

Carbohydrate mouth rinsing (CMR) has been shown to enhance exercise performance. However, the influence of CMR on repetitions to failure with different intensities (40% or 80% of 1 RM) is unknown. Therefore, the purpose of this study was to examine the effects of a 6% CMR solution on muscular endurance assessed at 40% and 80% of 1 RM in resistance-trained males. Sixteen resistance-trained males (age: 25 ± 3 years, height: 182 ± 6 cm, body mass: 86 ± 3 kg, body fat: 16 ± 3%, bench press 1 RM: 106 ± 16 kg, resistance training experience: 5 ± 1 years) completed four conditions in random order. The four conditions consisted of ten seconds of mouth rinsing with 25 mL solutions containing either maltodextrin or placebo (sweetened water) prior to performing a bench press muscular endurance test at either 40% of 1 RM or 80% of 1 RM. Total repetitions, heart rate (HR), ratings of perceived exertion (RPE), glucose (GLU) and felt arousal (FA) were recorded for each condition. There was a significant condition by intensity interaction (*p* = 0.02). CMR significantly increased total repetitions compared with placebo at the higher intensity (80% of 1 RM; *p* = 0.04), while there was no effect at the lower intensity (*p* = 0.20). In addition, HR, RPE, GLU and FA did not differ between conditions or across intensities (*p* > 0.05). In conclusion, CMR-enhanced muscular endurance performed at higher but not lower intensities.

## 1. Introduction

Carbohydrate (CHO) ingestion is an effective nutritional strategy to delay fatigue and increase exercise performance, particularly when the exercise duration is >90 min [1]. Mechanistically, CHO ingestion improves aerobic endurance by providing greater amounts of exogenous substrates for oxidative phosphorylation, in turn sparing muscle glycogen during prolonged high-intensity exercise [1]. However, carbohydrate mouth rinsing has been purported as an effective strategy to enhance various types of exercise such as endurance exercise (<1 h) [2,3], resistance exercise [4,5], repeated sprints [6,7] and neuromuscular performance [8]. Chambers et al. [9] have postulated that the ergogenic benefit of CHO mouth rinsing (CMR) is associated with the stimulation of brain regions implicated with the reward center and the regulation of motor activity via the exposure of taste receptors. Activation of these receptors leads to an improved central drive to the locomotor muscles. In addition, an increased motor-evoked potential amplitude, an index of potentiation of the brain-to-muscle neural pathway, increases corticomotor excitability, following CMR [10].

Although analytic [11] and systematic reviews [12] have concluded that CMR improves exercise performance, most of the beneficial effects occur during aerobic endurance exercise [13,14,15] while other modes of exercise such as resistance exercise are equivocal [16,17,18,19]. Methodological differences including testing protocols, training status, fed vs. fasting, duration and number of CMR used make direct comparisons difficult between studies and may alter the outcomes. Karayigit et al. [20] have reported that low (6%), moderate (12%) and high (18%) doses of CMR did not enhance bench press 1 repetition maximum (1 RM) strength or muscular endurance performed at 40% of 1 RM to failure in females. The authors have suggested that the lack of effect of CMR was not related to the concentration. In contrast, Clarke et al. [4] revealed that a 6% CMR solution (prior to each exercise test) significantly improved bench press and back squat repetitions to failure at 60% 1 RM. The same research group also demonstrated no beneficial effects of a 6% CMR solution on bench press 1 RM strength and repetitions to failure at 60% 1 RM [21]. However, in the aforementioned study, CMR was utilized at one time point before both the 1 RM strength measurement and muscular endurance test, a period which would include a time gap of ~10 min between rinsing and the muscular endurance test [21]. The authors note that for future studies, extra rinses before the muscular endurance test may be warranted to have a greater effect [21].

Mechanistically, there are two purported possibilities to explain the beneficial effects of CMR on muscular performance. First, CMR decreases ratings of perceived exertion (RPE) during exercise due to activation of brain areas associated with reward, pleasure and sensory perception (orbitofrontal cortex and striatum) [9]. Second, CMR is likely to enhance the excitability of the corticomotor pathway [10] and decrease neuromuscular fatigue [22]. Neuromuscular fatigue is suggested to result from an attenuation in central motor output and motor neuron activation [23]. Central and peripheral fatigue is also shown to be task-specific and vary between high- and low-intensity exercise [24]. Specifically, using lower intensities was suggested to cause greater fatigue compared with higher intensities which may alter the effects of CMR during resistance exercise [24]. Krings et al. [17] have also suggested that potential thresholds exist for enhancement with CMR, since the neuromuscular system may not be able to compensate for the high degree of fatigue accumulated during resistance exercise. In this regard, divergent results in resistance exercise responses to CMR are likely to be associated with intensity of the muscular endurance tests. All [19,20,25,26] but one [27] of the studies that examined the influence of CMR at 40% of 1 RM did not detect any benefit from CMR. Similarly, studies using an intensity of 60% [21,28] and 70% [17,18] of 1 RM did not report any benefit of CMR. On the other hand, Decimoni et al. [5] and Bastos-Silva et al. [16] demonstrated that CMR improved repetitions to failure performance at 75% and 80% of 1 RM, respectively. Since a high degree of localized, peripheral fatigue, induced by low-intensity resistance exercise, was proposed to exceed the capacity of any central effect of CMR [28], one can assert that as the test intensity decreases, so do the effects of CMR. However, no single study to date has investigated different intensities on the ergogenic potential of CMR. Therefore, the aim of this study was to investigate the effects of CMR on 40% (low) and 80% (high) of 1 RM repetitions to failure. Since greater fatigue is predicted to accumulate during low-intensity resistance exercise compared with high-intensity exercise [17,24,28], we hypothesized that CMR would enhance 80% but not 40% of 1 RM repetitions to failure.

## 2. Materials and Methods

### 2.1. Participants

Sixteen healthy, resistance-trained males volunteered to participate (age: 25 ± 3 years, height: 182 ± 6 cm, body mass: 86 ± 3 kg, body fat: 16 ± 3%, bench press 1 RM: 106 ± 16 kg, resistance training experience: 5 ± 1 years). A priori power analysis suggested a sample size of fifteen participants was necessary to detect a difference between conditions given an estimated effect size of 0.4, a 1–β error probability of 0.95 and an alpha value of <0.05. All participants declared that they had not used any ergogenic aids that may alter muscular biology and performance within three months from the start of the study. Participants had at least 5 years of resistance training experience, training at least four times per week for the previous year with training that must have included upper body exercises such as bench press. Criteria for inclusion in the current research included being (a) between 19–30 years of age, (b) having a resistance-trained classification (performing moderate to high intensity upper body resistance exercise 2–3 times per week for at least 6 months [29], (c) having no neuromuscular and musculoskeletal disorders, and (d) being able to lift successfully a load in bench press equal to 100% of their current body mass. This study was conducted in accordance with the Declaration of Helsinki, was approved by Muş Alparslan University Ethic Committee (24617-10/21) and participants were made aware of the purpose and potential risks of participation before providing written informed consent.

### 2.2. Study Design

**Figure 1:**
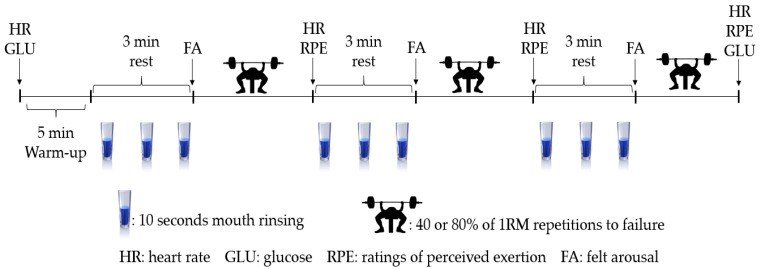
Schematic diagram of experimental protocol.

A repeated measures, randomized, double-blind, counter-balanced, cross-over design was used. After determination of bench press 1 RM and two familiarization trials, participants visited the laboratory on four separate occasions (in random order): 40% of 1 RM bench press repetitions to failure with a CMR solution or placebo (PLA), and 80% of 1 RM bench press repetitions to failure with a CMR solution or PLA. Experimental visits took place on different days (separated by 72 h) and in the morning between 8.00 and 9.00 am after an overnight fast (12 h). Each participant completed their respective experimental sessions at the same time of day to avoid any moderating effect of circadian rhythm. Participants warmed up for 5 min on a treadmill and lifted 20 kg of weight 10 times followed by 3 sets of bench press, interspersed by a standardized 3 min of passive recovery. Heart rate (HR) (Polar Team 2 telemetric system, Kemple, Finland), 6–20 ratings of perceived exertion (RPE), felt arousal (FA) [30] and capillary glucose (GLU) (Accutrend Plus, Roche Diagnostics, Mannheim, Germany) were recorded at different time points throughout the experiments. Participants were required to abstain from the ingestion of caffeine for at least 12 h before all testing sessions and to not perform vigorous physical activity in the 48 h leading up to each laboratory visit. Each participant recorded their 24 h dietary intake before the first testing session and were asked to replicate this diet before subsequent sessions. Test procedures are summarized in Figure 1.

### 2.3. One Repetition Maximum Bench Press Strength (1 RM)

Bench press 1 RM was determined in the first familiarization trial using standard procedures [20,25,29]. Briefly, participants conducted a progressive warm-up with a self-selected light to moderate weight prior to attempting to lift estimated near-maximal loads. Following a short passive rest, the resistance was increased by 5–10% and a single lift attempted. If the 1 RM attempt was performed successfully in each step, then weight was increased by 5–10%. If the lift was unsuccessful, the weight was decreased by 2.5–5% for another 1 RM attempt after three minutes of passive rest. This procedure was repeated a further 2–3 times and the highest successful attempt was recorded as the 1 RM of participant.

### 2.4. Bench Press 40% and 80% of 1 RM Repetitions to Failure

Muscular endurance performance was tested at either 40% (low intensity) or 80% (high intensity) of 1 RM repetitions to failure. Repetition number was recorded during 3 sets of bench press exercises with 3 min of passive recovery. Repetitions to failure protocol was performed on a bench press rack with safety bars in place (Esjim, Eskişehir, Turkey) and in the presence of a qualified spotter. Bar grip position was recorded for each participant and kept constant for the subsequent sessions. Participants were asked to keep their buttocks on the bench press rack and their heels touching the floor for each repetition to standardize bench press exercise technique. A successful repetition was defined as lowering the bar until touching the chest and then raising the bar so elbows were fully extended. Repetition tempo was standardized via a metronome (2 s for both eccentric and concentric phases). Testing was terminated based on three criteria; (1) deterioration of bench press exercise technique and posture; (2) inability to follow the rhythm of metronome for three consecutive repetitions; and (3) voluntarily exhaustion.

### 2.5. Mouth Rinsing Protocol

During each laboratory visit, participants were given a 25 mL bolus of either 6% maltodextrin or a water placebo solution during each intensity condition. Three hundred mg of sucralose was dissolved into each solution. Each solution was delivered in a plastic cup and was rinsed in the buccal cavity for 10 s each min (3 times) between sets in the bench press muscular endurance test (Figure 1). A research assistant unrelated to the research protocol coded and prepared the solutions using electronic laboratory scales with one milligram of sensitivity at room temperature.

### 2.6. Statistical Analysis

Data are reported as mean ± SD, with an alpha level of *p* < 0.05. All of the statistical analyses were carried out using IBM SPSS statistics (version 22.0; IBM Corp., Armonk, NY, USA). Normal distribution was confirmed with a Shapiro–Wilk test. A two-way repeated measured analysis of variance (ANOVA) was performed to analyze main effects for (1) condition, (2) test intensity, (3) time or set and (4) condition × test intensity × time or set interaction. Mauchly’s test analyzed the sphericity assumption followed by the Greenhouse–Geisser adjustment if required. If a significant interaction or main effects was detected, pairwise comparisons with Bonferroni’s corrections were applied. The effect sizes were calculated using partial eta squared (η^2^) for each repeated-measures analysis of variance as trivial (<0.10), moderate (0.25–0.39), or large (≥0.40). Intraclass correlation coefficients (ICC) were assessed to determine the consistency of the four trials across conditions (two-way mixed model in consistency type).

## 3. Results

### 3.1. 40% or 80% of 1 RM Muscular Endurance Performance

**Figure 2:**
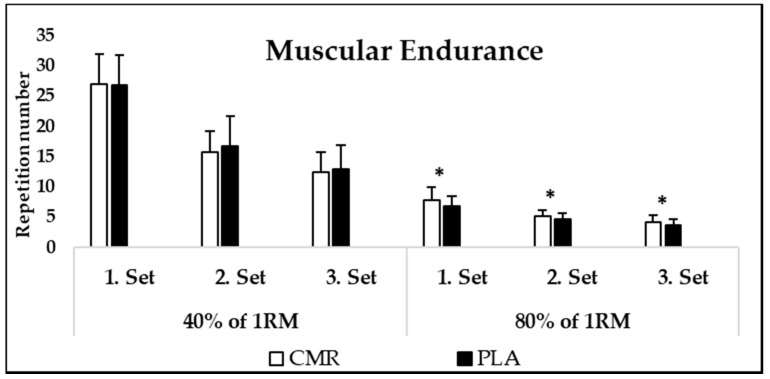
Mean (SD) bench press 40% or 80% of 1-RM repetitions to failure performance. CMR: carbohydrate mouth rinsing; PLA: placebo; *: Significantly different from PLA.

**Figure 3:**
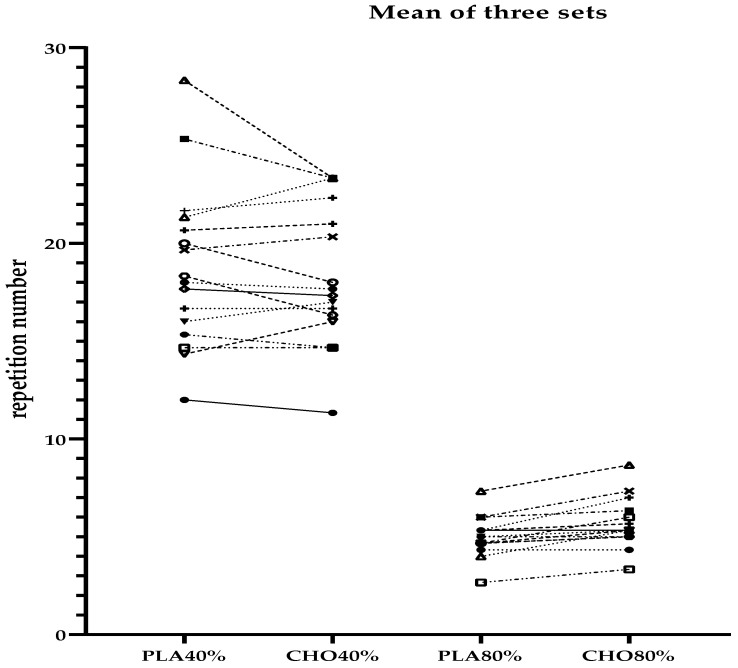
Individual data of mean of three sets of bench press endurance test. PLA40%: placebo rinse at 40% 1 RM endurance; CMR40%: carbohydrate rinse at 40% 1 RM endurance; PLA80%: placebo rinse at 80% 1 RM endurance; CMR80%: carbohydrate rinse at 80% 1 RM endurance.

There was no main effect for condition (*p* = 0.58, η^2^ = 0.10). Further, no condition × set (*p* = 0.39, η^2^ = 0.06) or condition × intensity × set interaction (*p* = 0.81, η^2^ = 0.01) was detected in muscular endurance test performance. However, condition × intensity interaction was observed to be significant (*p* = 0.02, η^2^ = 0.16). Post hoc analysis revealed that there was no difference between CMR40% and PLA40% conditions (*p* = 0.20). Mean repetition number during three sets of bench press endurance test was 18.75 in PLA40% and 18.33 in CMR40%. However, CMR80% was significantly higher than PLA80% condition (*p* = 0.04). Participants completed 5.64 repetitions in the CMR80% and 4.97 repetitions in the PLA80% conditions during three sets of bench press endurance test. Post hoc analysis also showed that CMR80% was significantly higher during the first (*p* = 0.011), second (*p* = 0.006) and third set (*p* = 0.001) compared with PLA80% (Figure 2 and Figure 3). ICC values ranged between 0.87–0.94 during both the 40% and 80% of 1 RM endurance tests.

### 3.2. Heart Rate, RPE, Felt Arousal and Glucose

HR did not differ between conditions (*p* = 0.51, η^2^ = 0.04) and no condition × time interaction was detected (*p* = 0.51, η^2^ = 0.05). However, as expected, HR values significantly increased over time (*p* = 0.01, η^2^ = 0.93) through the testing protocol. There was no condition × time interaction (*p* = 0.13, η^2^ = 0.11) for RPE. In addition, a main effect for condition was not significant (*p* = 0.91, η^2^ = 0.01). Felt arousal was also not different between conditions (*p* = 0.30, η^2^ = 0.07). Condition × time interaction was also not significant (*p* = 0.47, η^2^ = 0.05). However, felt arousal values decreased significantly over time (*p* = 0.01, η^2^ = 0.45). Lastly, glucose levels were not different between conditions (*p* = 0.85, η^2^ = 0.01). Condition × time interaction was also not significant (*p* = 0.31, η^2^ = 0.07). However, glucose levels were significantly higher at the post-test compared to pre-test time points (*p* = 0.01, η^2^ = 0.45) (Table 1).

## 4. Discussion

The novel finding of the current study is that CMR increased 80% but not 40% of 1 RM repetitions to failure during a bench press exercise. However, CMR did not affect heart rate, RPE, felt arousal or capillary glucose. These findings support our hypothesis that CMR may have a significant impact on 80% but not on 40% of 1 RM repetitions to failure.

Painelli et al. [18] have reported that CMR did not increase bench press repetitions to failure. The authors have suggested that the high test intensity (70% of 1 RM) used in the test protocol may have affected the results [18]. In support, Clarke et al. [21] have highlighted that when the load elicits near maximal heart rate and RPE values, it creates a “ceiling effect” which reduces the possibility of detecting subtle ergogenic effects during resistance exercise. In this regard, Clarke et al. [21] have suggested that future research use a low intensity (<60%) of 1 RM in the test protocol. This speculation has not been confirmed by previous results [20,25] and the current study shows that CMR did not enhance the performance of low (40%) intensity 1 RM repetitions to failure. Similar to our results, significant improvements have been observed with CMR for 1 RM bench press endurance test at 80% [16]. On the other hand, Clarke et al. [21] found no influence of a 6% CMR solution on 60% of 1 RM endurance and suggested that future research should increase the number of CHO rinse exposures before a muscular endurance test. It appears that this suggestion has worked during 80% of 1 RM endurance test in our study. We used a repeated (three times) CMR protocol. This is the most important difference between our study and previous studies that used both high test intensities (70–75% of 1 RM) and only a single rinse (10–15 s) of carbohydrate solution and in which no positive results were reported [17,18]. Total exposure duration to CHO before each set was 30 s in our study. In support, Phillips et al. [7] were the first to show that serial administration (8 × 5 s = 40 s in total) of a 6% CMR significantly improved peak power output during a cycle sprint. In addition, 10 s of CMR was superior to 5 s during 30 min of self-paced cycling performance [31]. In contrast, high-intensity sprinting [32] and a Yo-Yo intermittent recovery test [33] performance did not increase with serial rinsing of CHO (30 and 80 s, respectively). Previously, three studies [20,25,26] from our laboratory observed no improvement in 40% of bench press 1 RM repetition to failure performance with serial CHO rinsing (20 or 30 s). One of the reasons for not detecting improvements in the 40% of 1 RM endurance can be that oral receptors may need more CHO rinse time (>30 s). In support, ergogenic magnitude of CMR on sprint [7] and aerobic endurance [31] performance was demonstrated to have relationship with exposure duration and number of CMR. However, no study, to date, has investigated the optimal rinse time and/or duration during resistance exercise, thus a direct comparison cannot be made.

Potential ergogenic benefits of CMR during endurance-based exercise range between 1–3% [2,3,11]. In the current study, CMR (16.93) increased mean repetition number during 3 sets at 80% of 1 RM endurance test by 13% compared with placebo (14.93). Conversely, despite a non-significant effect, mean repetition number during 3 sets at 40% of 1 RM endurance test was 2% higher even in placebo (56.23) than the CMR (54.99) condition. ICC values at 40% of 1 RM endurance test, as a consistency index, were between 0.87 and 0.94 which means high repeatability during the four test conditions. However, the mode of current and previous [19,21] test protocols may be arguable as they may not be sensitive enough to reveal subtle effects of CMR at low intensities of resistance exercise. Clarke et al. [21] and Painelli et al. [18] have purported that expected benefits of CMR (+2–3%) may be lost due to large inter-day strength variations which have been shown to be 5% [34]. This may not be the case in our study because ICC values showed high consistency. On the other hand, independently of the sweetness, both 7.1% glucose and 6.4% maltodextrin solutions enhanced isokinetic performance as shown by the greater total amount of work achieved [35]. Further studies are required to investigate the influence of CMR on strength performance using more sensitive measuring devices such as isokinetic dynamometer or gauge.

In the current study, divergent ergogenic responses to CMR between low (40%) and high (80%) intensity muscular endurance performance can be related to the magnitude of fatigue [23,24]. An inverse relationship between the resistance exercise test intensity and the magnitude of central fatigue has been reported by Hunter [24]. In support, using lower contraction intensities at 20% and 30% of MVC caused two to three times greater reductions in voluntary activation levels (VA%) compared with intensities of 80% and 75%, respectively [36,37]. Further, repetition to failure is characterized by the ability of the muscle to maintain contractions perpetually against a given percentage of 1 RM, which is related to the ability of the central nervous system to drive neural impulses to the fatigued muscles [38]. Our 40% of 1 RM test protocol possibly induced too much of a reduction in neural drive at a level which cannot be rescued by CMR. In this regard, we can allege that a high degree of central and localized peripheral fatigue induced by our 40% of 1 RM endurance test protocol may have exceeded the ability of CMR to exert its central effect. This assertion is also supported by Green et al. [28] and Krings et al. [17] whose studies showed no effect of CMR during resistance exercise because of a high degree of accumulated fatigue that cannot be overcome by CMR. In contrast, it has been shown recently that CMR does not attenuate the decline in maximal isometric strength or voluntary activation % after evoking different levels of central fatigue with low (20%) and high (80%) intensity exercise [39]. However, it is important to note that the aforementioned study was conducted in a fed state which may decrease the ergogenic magnitude of CMR [2]. In addition, Jensen et al. [40] have observed that CMR attenuated the decline in knee extension torque with peripheral mechanisms rather than through the central influence of CMR. To test this suggestion, Black et al. [41] used an interpolated twitch technique (ITT) to measure the central and peripheral contributions to force loss and reported that CMR did not enhance strength or voluntary muscle activation. In contrast, Bazzuchi et al. [35] concluded that repeated CMR could counteract fatigue-induced decline in neuromuscular performance of elbow flexors, pointing out that CMR may activate positive afferent signals able to modify motor output. In support, an increased central motor drive during cycling exercise with CMR has been suggested to be the main influencing mechanism [8,13]. Because we did not use ITT or EMG analysis in the current research design, firm conclusions cannot be made regarding which of the peripheral or central fatigue pathways may moderate the positive influence of CMR during 80% of 1 RM endurance test.

It may have been reasonable to expect a preferential effect of a CMR on RPE values because of its stimulatory influence on brain regions associated with reward and motivation [9]. However, RPE values in the current study did not change, something which is common in the literature [4,17,18,19,20,21,25,26,28], although with a few exceptions [5,13]. Heart rate, capillary glucose and felt arousal values were also not affected by CMR. Our results are in accordance with previous studies [3,17,19,21,25]. Clarke et al. [21] explained the lack of difference in heart rate and RPE with reference to a “ceiling effect” created by the high intensity exercise. However, the highest heart rate (137 bpm) and RPE (17) values are not near maximal, thus, a “ceiling effect” does not appear to be justified.

One limitation of the current study was that a no-rinse control was not included in the design, something which gave rise to an underestimation of the “true” effect of the intervention. If a no-rinse control condition had been used, it is then reasonable to expect that CMR during 40% of 1 RM endurance may increase performance according to a control condition [42]. Further, blinding effectiveness was not measured and the “expectancy” phenomenon may affect the outcomes of the current study. We also did not measure readiness to invest effort, because greater concentration on each repetition results in greater motor unit recruitment and voluntarily improves the activity of a muscle group [43]. Perhaps our participants were more motivated or concentrated on the 80% of 1 RM endurance test because of the higher intensity compared with the 40% of 1 RM protocol. Moreover, the inability to measure EMG or VA% during bench press exercises make it difficult to fully elucidate exact mechanisms of CMR on 80% of 1 RM endurance. Lastly, although conducting tests after overnight fasting can potentiate benefits of CMR, strength training is not commonly performed in a fasted state.

## 5. Conclusions

Mouth rinsing three times with a 6% carbohydrate solution can improve the performance of 80% 1 RM repetitions-to-failure performance possibly via centrally mediated mechanisms [10]. However, the same benefit was not observed during a lower intensity (40% of 1 RM) protocol. Coaches and athletes should use CMR strategically to optimize high-intensity muscular endurance. Future research is warranted to determine whether these acute alterations persist over a training program.

## Figures and Tables

**Table 1:** Heart rate, RPE, felt arousal and glucose values.

	PLA40%		CMR40%		PLA80%		CMR80%	
	M	SD	M	SD	M	SD	M	SD
Heart Rate (Beats/min)								
Pre-test	64.93	7.91	64.37	6.42	64.62	6.50	63.18	6.18
1. set	129.18	20.66	126.37	20.74	128.62	20.33	133.93	21.54
2. set	129.62	22.10	129.56	20.41	132.87	20.47	135.00	18.30
3. set	133.00	24.58	133.31	23.36	135.00	21.25	137.68	20.44
Ratings of Perceived Exertion (RPE) (6–20)								
1. set	14.12	3.15	14.06	2.83	14.43	2.63	14.50	3.42
2. set	16.68	1.95	16.12	1.66	16.06	1.80	15.75	2.64
3. set	17.43	1.86	17.62	1.74	17.00	1.59	16.87	2.75
Felt Arousal (1–6)								
1. set	4.31	1.07	5.00	0.81	4.50	1.31	4.62	0.95
2. set	4.00	1.09	4.06	1.06	4.00	1.15	4.12	0.95
3. set	3.56	1.20	3.87	1.25	3.93	1.12	3.87	1.14
Glucose (mg/dL)								
Pre-test	81.18	14.00	78.68	15.75	82.75	10.68	88.06	13.12
Post-test	88.37	8.36	88.43	6.93	88.56	7.56	86.87	15.29

PLA40%: placebo rinse at 40% 1 RM endurance; CMR40%: carbohydrate rinse at 40% 1 RM endurance; PLA80%: placebo rinse at 80% 1 RM endurance; CMR80%: carbohydrate rinse at 80% 1 RM endurance; pre-test: immediately prior to test protocol; post-test: immediately after test.

## Data Availability

The data presented in this study are available on request from the corresponding author. The data are not publicly available due to restrictions privacy.
